# The nasal cantilever technique in children undergoing primary cleft lip surgery: novel concepts and review

**DOI:** 10.1515/iss-2020-0016

**Published:** 2020-09-07

**Authors:** Badr M. I. Abdulrauf

**Affiliations:** Section of Plastic Reconstructive Surgery, Department of Surgery, King Faisal Specialist Hospital and Research Center Jeddah, Jeddah, Saudi Arabia

**Keywords:** cleft, correction, deformity, lip, nasal, rhinoplasty

## Abstract

**Background:**

Nasal deformity associated with cleft lip deformity is a challenging issue, encompassing controversies, theories, and a diversity of techniques. Historically, esthetic outcomes have ranged from being below expectations to barely acceptable.

**Method:**

Based on the concept that the nasal cartilaginous framework in clefts is similar to that of a collapsing pyramid, a novel suspension technique has been described. The entire cartilaginous structure is lifted from the infratip segment with a loop suture and is secured in a cantilever fashion onto the periosteum overlying the nasal bone. This part of the operation is performed in a semiclosed manner. The technique is applied during primary surgery in bilateral and unilateral nasal cleft lip deformities, with changes in the orientation of the cantilever loop suture. Studies conducted by Masters S. Tajima, H. McComb, H. Thomson, D. Fisher, and J. Mulliken, which are most relevant to this article, have been reviewed and discussed throughout.

**Results:**

The technique was first applied over 10 years ago. A case series of nine children whose parents consented to the developing technique is presented with follow-up ranging from months to years, along with technical descriptions and illustrative drawings. None of these cases had preoperative orthopedic correction, molding, or postoperative nostril splints. The esthetic outcome was optimal enough; none of the cases requested a secondary correction.

**Conclusion:**

The nasal cantilever technique is a novel concept in cleft nasal deformity, which can be used in conjunction with an appropriate lip technique, per the surgeon’s discretion. Other than a learning curve, we believe that it provides a solid correction by securing the cartilaginous structures after they have been mobilized to a stable base, the nasion.

## Introduction

With experience, one realizes the difficulty in achieving good results for the nose in cleft lip cases. Closing a large cleft and achieving good alignment of the lip is usually challenging for a junior surgeon. It has been truly stated that cleft lip surgery is essentially an operation to the nose [1].

Unilateral cleft lip nasal deformity (UCLND) has been investigated and written about far more than its bilateral counterpart, an observation that can easily be made upon reviewing this subject [2], [3], [4], [5]. The rationale is not limited, and the solutions applied to the former can simply be applied to both sides. The tilt of the tripod in the unilateral cases begins with the infrastructure (maxilla) up to the skin; hence, the asymmetry has challenged surgeons the most [5].

We introduce a complementary concept that combines suspension and traction of the freed up and fully mobilized lower lateral cartilages, however, to the nasal bone’s periosteum and in a closed manner. This is besides a few well-established maneuvers.

Although first thinking of and applying this idea over 10 years ago, we only recently decided to report it after observing convincing results ourselves. More importantly, parents of these children have not asked for further nasal correction, unlike some other cases that have undergone preschool cleft rhinoplasty. The other factors common in these particular cases were that none of them had any presurgical orthopedic manipulations, nasoalveolar molding, or postoperative nostril splints.

Reports for nine children subjected to our technique are presented in this paper. They were selected on the basis of delayed timing of surgery and/or their parents fully consenting to the novel innovative technique. The first two cases are presented mainly for technical demonstration purposes.

## Method/operative technique

The marking and operation are performed under loupe magnification. Besides diluted adrenaline, the nasal mucosa is also infiltrated with injectable normal saline, specifically to the tip and columellar area, if present, and over the lower lateral cartilages. This step helps in hydrodissection, making isolation of the flimsy cartilages relatively easy.

Surgery begins with lip incisions and dissection; the specific method selected depends on the case. Then, attention is turned toward the nasal operation.

**Figure 1:**
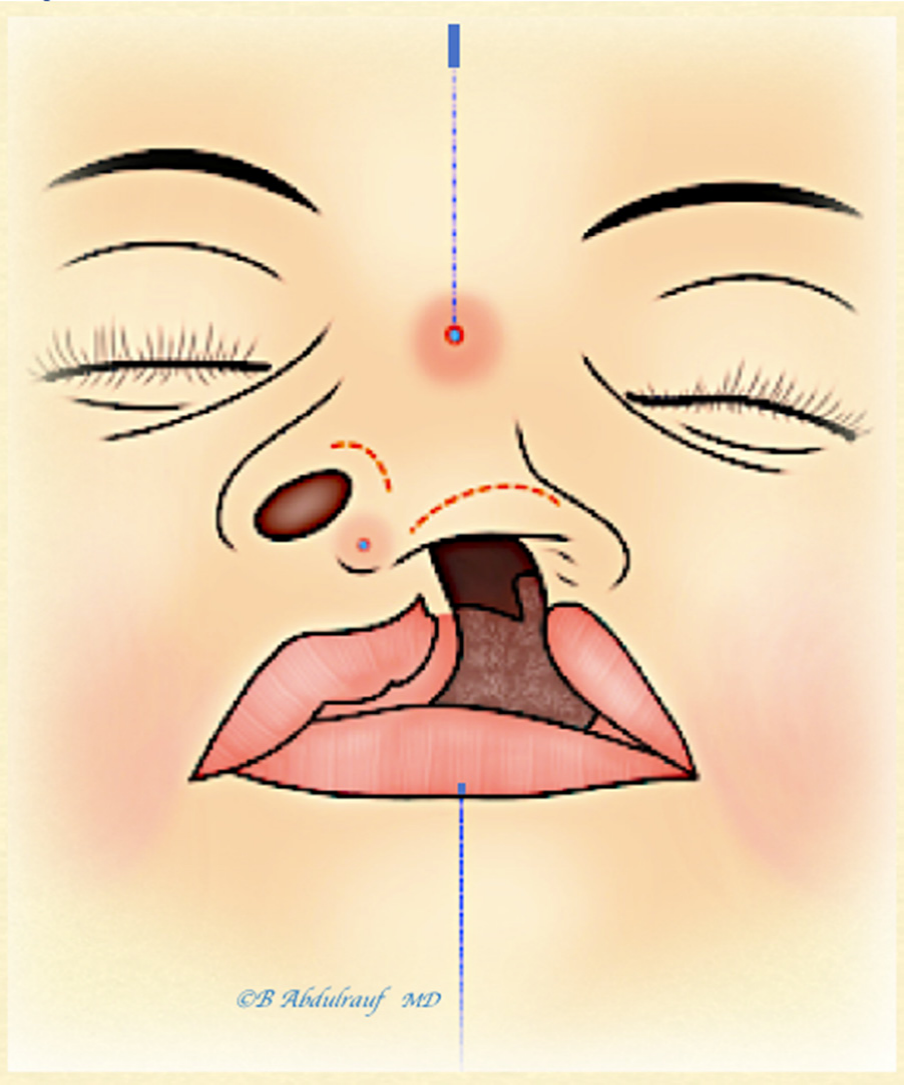
Incisions utilized in the semiclosed nasal cantilever technique (NCT). These include bilateral rim incisions and two external 18 G needle-induced stab incisions, a cephalic one at the radix and a caudal one at the future infratip point. A “UCLND” example is used in this illustration. Midline is marked on the glabella and on the lower lip as a reference point for the cephalic radix opening, where the nasal tip should be aimed to be positioned at completion of the case. UCLND, unilateral cleft lip nasal deformity.

Nasal surgery is performed entirely through rim incisions and two external 18 G needle-induced stab incisions (Figure 1).

**Figure 2:**
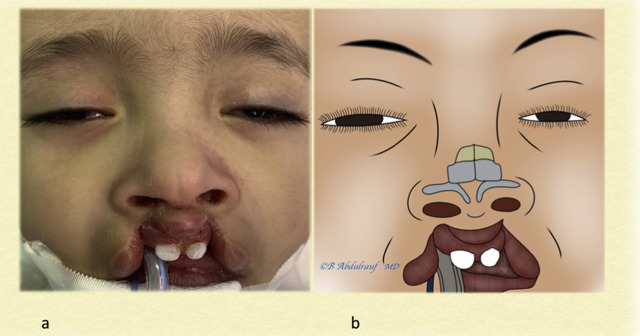
Case 1: (a) BCLND, splayed lower lateral cartilages, flattened bifid tip, and short columella, and (b) artist’s depiction of underlying anomaly. BCLND, bilateral cleft lip nasal deformity.

Initially, rim incisions are made. The alar cartilages are carefully dissected and fully skeletonized on both sides using a closed technique. In UCLND, dissection on the normal side is limited to the dome and proximal lateral crus. Nasal skin is undermined all the way to the radix, including the triangular cartilage region. Lateral crural steal or mobilization is considered to build up the deficient columella. Transdomal equalization sutures are placed using Polydiaxone, J&J (PDS) 5.0. Next, a cinch suture is placed in the fibrofatty tissue using Ethibond 3.0 (Ethicon, Somerville, NJ) (holding both alae in case of bilateral cleft lip nasal deformity or one side in case of UCLND) and is secured in the premaxillary periosteum. The domal and cinch sutures are both left untied at this point.

Two 18 G needle-induced stab incisions are made, first on the nasal radix and second just caudal to the future nasal tip.

**Figure 3:**
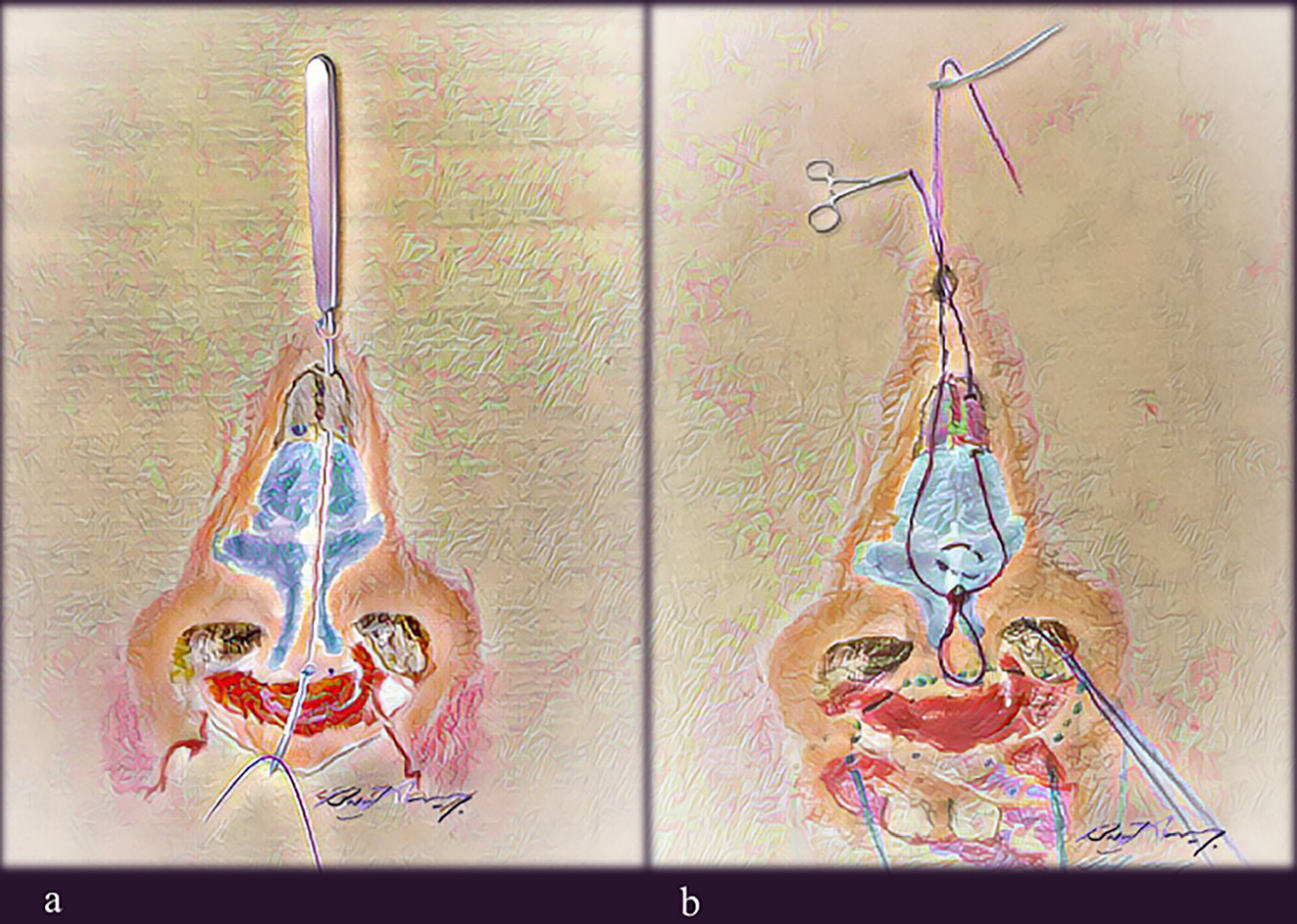
Author’s illustration of the NCT in a case of BCLND; a malleable suture passer has been introduced from radix opening cephalically, partly embedded in the periosteum overlying the nasal bone, exiting from the infratip opening caudally. (a) It catches the thread (violet color) to retrieve it and pull it out at the radix. Then, after repeating the same maneuver on the other side but maintaining a superficial track, a loop is created. (b) The tip transdomal suture (blue) and cinch suture (green) are also shown; none of the three key sutures are tied yet. BCLND, bilateral cleft lip nasal deformity; NCT, nasal cantilever technique.

A malleable suture passer is introduced through the mini stab incision at the radix, which is then advanced subcutaneously in the previously undermined plane on one side of the cartilaginous framework and brought out from the infratip needle-induced incision. A Vicryl 4.0 (Ethicon, Somerville, NJ) suture is used and is caught and brought out of the opening at the radix. The suture passer is then reintroduced from the radix, this time piercing the nasal bone periosteum. It is then driven into the contralateral side in relation to the cartilaginous framework. It is brought out again through the infratip opening and the other end of the thread is held and pulled back smoothly, making sure not to lose the subperiosteal plane of the nasal bone. In this manner, a loop is created that goes around the infratip (caudal to the domes) and holds the entire cartilaginous framework to the nasion. Catching the periosteum on either the first or second pass is optional; either way, the objective is to make the final surgical knot in the nasal bone periosteum (Figures 3, 10).

**Figure 4:**
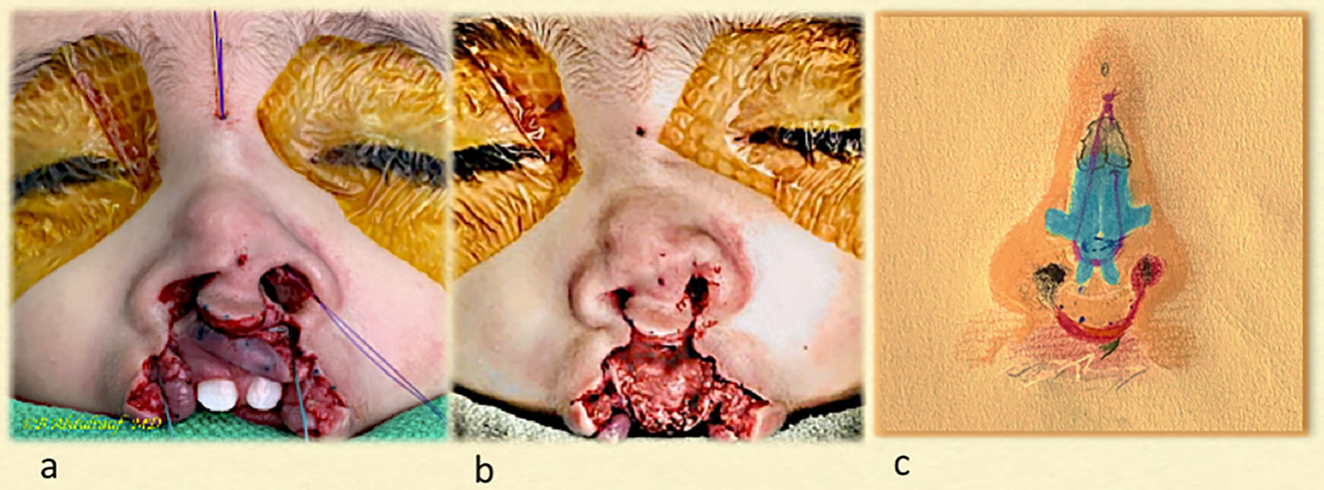
Case 1: (a) Intraoperatively, all three main sutures have been placed as per the steps shown in the previous illustration. The sutures have been tied beginning with the cinch, followed by the transdomal, and lastly the loop suture was tied. (b) The bilateral cleft lip is not repaired yet, but the nose has been fully addressed with the semiclosed technique. (c) Author’s depiction of the underlying nasal repair and role of various sutures.

**Figure 5:**
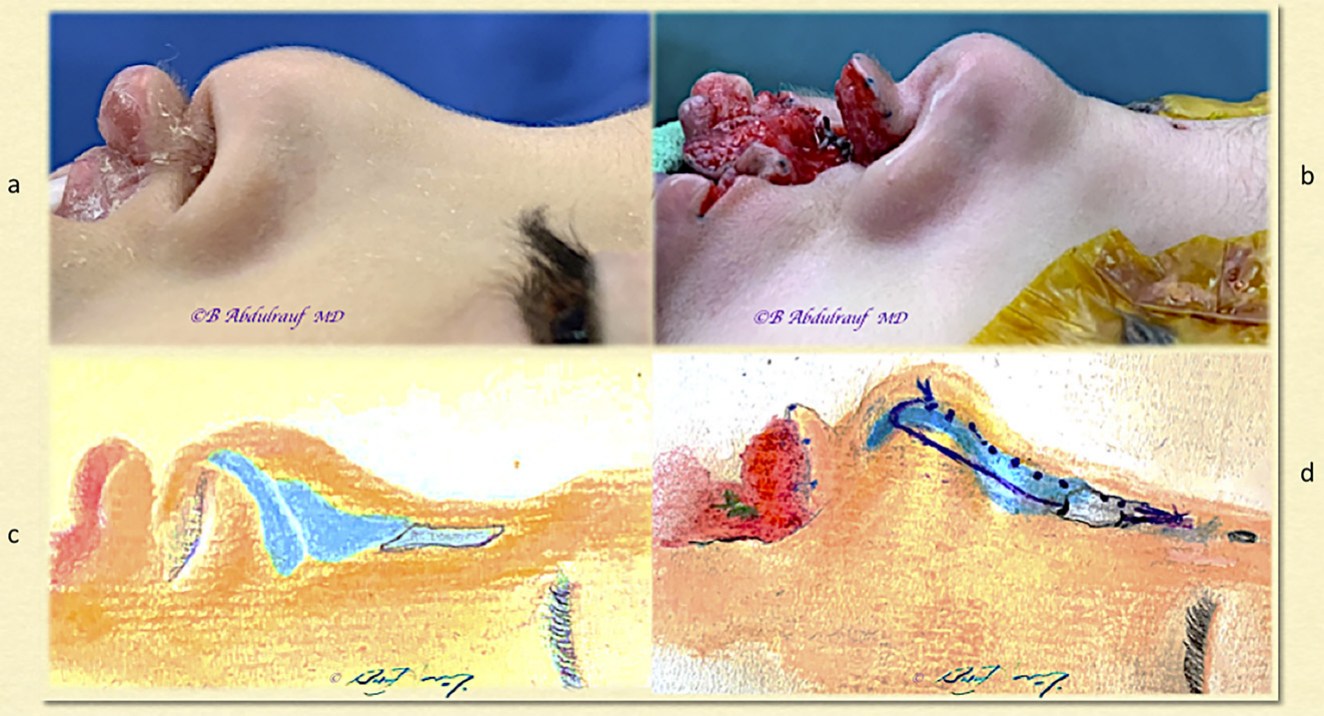
Case 1: (a, b) Profile views intraoperatively and immediately after completing nasal correction using the NCT; the lip is still open. Author’s illustration of the underlying repair in profile view, which provides the best explanation of how the technique suspends the entire nose from the infratip region and holds it solidly with the mattress cable suture tied to the periosteum. (c, d) The columella has been restored, its shape maintained with the loop suture as a checkrein mechanism. NCT, nasal cantilever technique.

**Figure 6:**
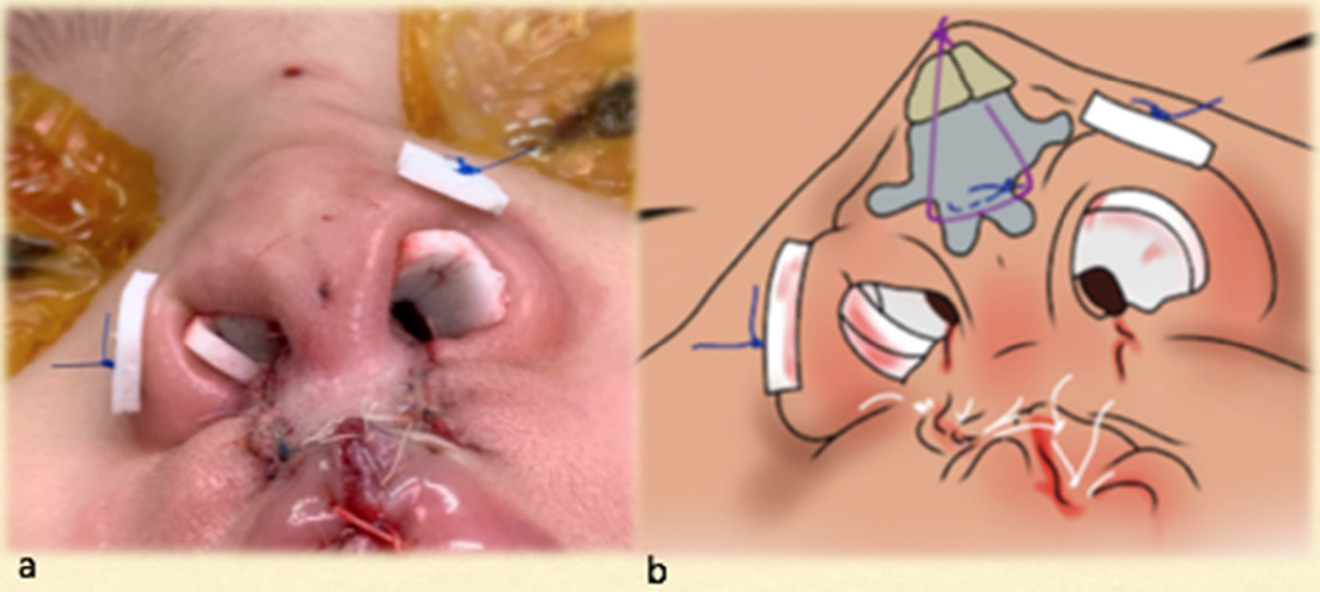
Case 1: (a) Basal view at completion; bolster sutures are used occasionally to reduce minor hematomas and fibrosis. (b) Author’s view of the underlying repair.

Next, the cinch suture is tied, followed by the transdomal equalization suture(s). The loop suture is then tied, and the knot is kept well away from the radix opening (Figures 4–6, 11). No septal interventions were considered in this technique.

**Figure 7:**
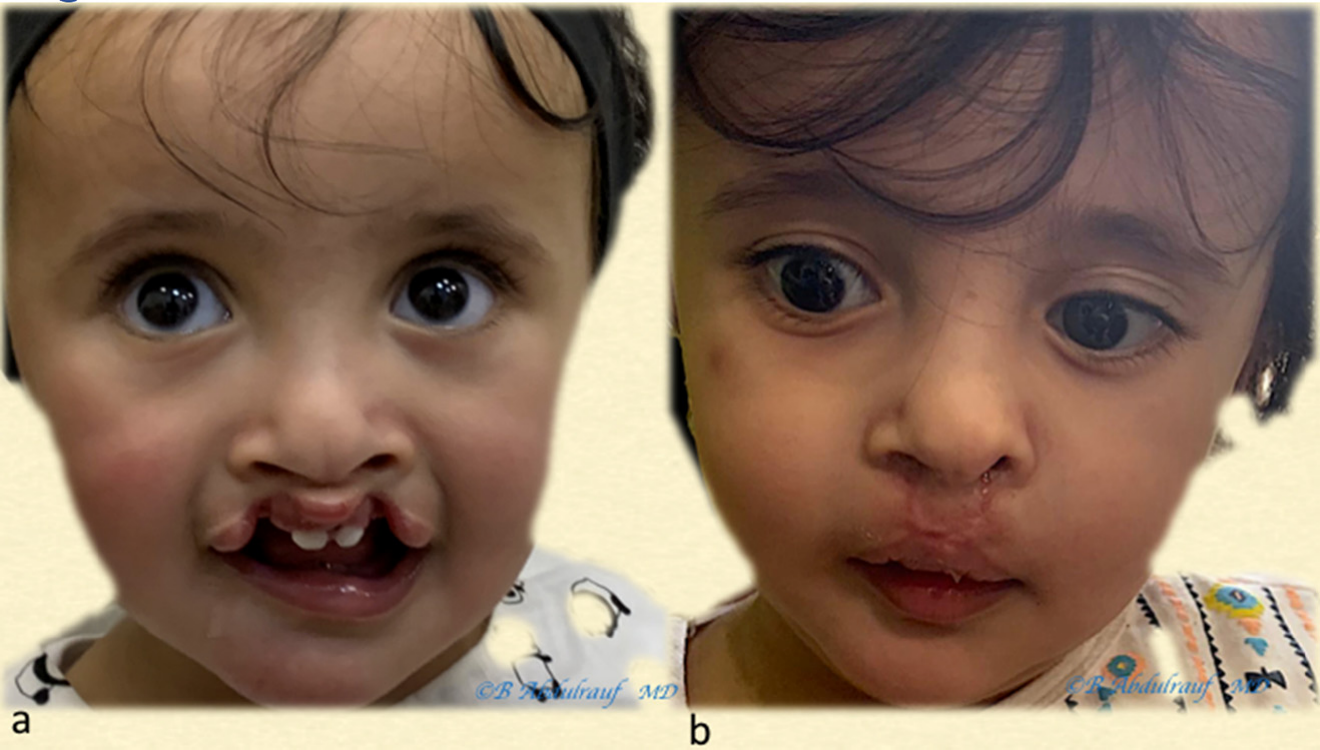
Case 1: (a) Used for technique demonstration as an example of BCLND, operated at 14 months. (b) At the three-month follow-up, we intentionally preserved the philtral width at primary surgery instead of banking or discarding. This would be extremely useful in case of a future rhinoplasty; otherwise, the lip can be revised to optimal philtral width at teenage. BCLND, bilateral cleft lip nasal deformity.

**Figure 8:**
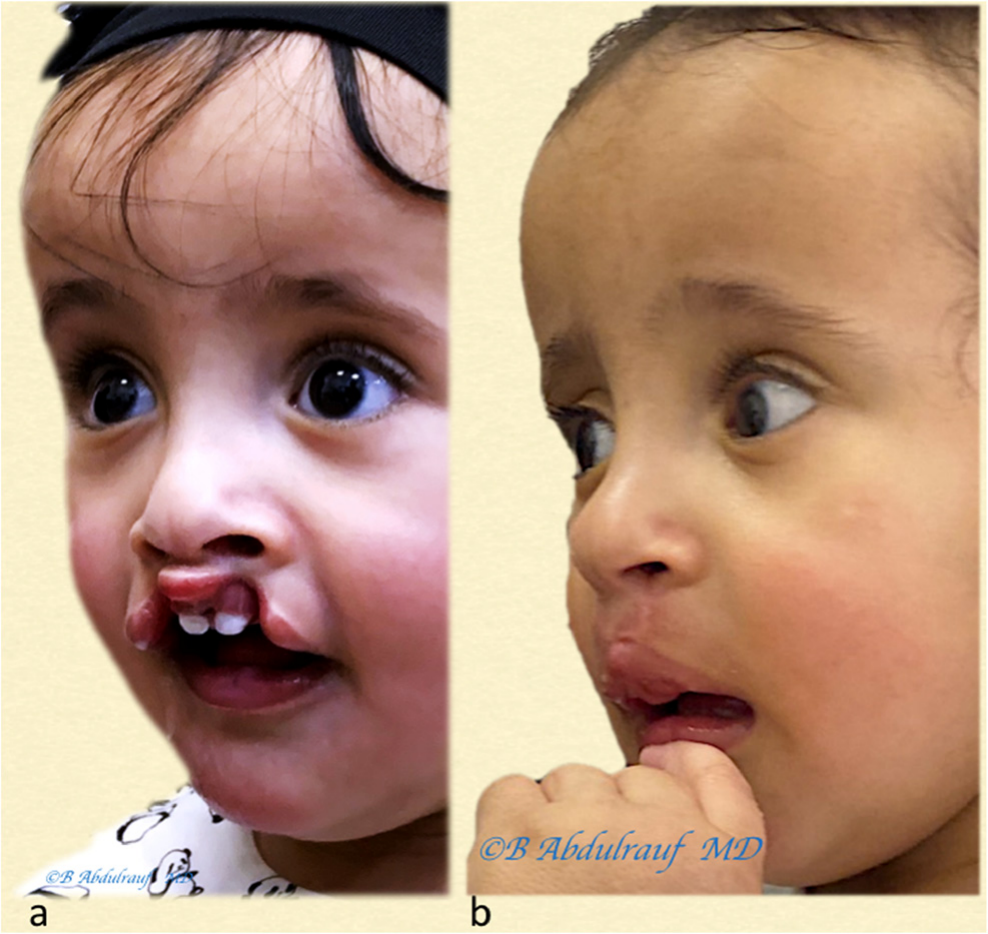
Case 1: (a) Peopertaive (b) follow up six months postoperatively. The columella has been lengthened, and the nasal tip is defined and repositioned.

**Figure 9:**
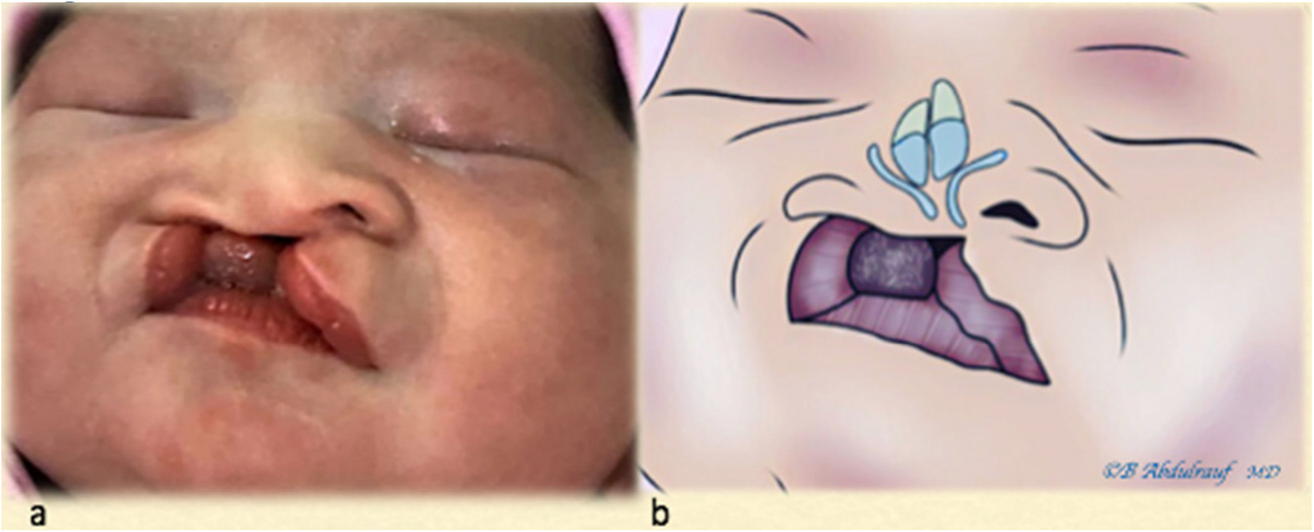
Case 2: (a) As a newborn with severe UCLND, there is a significant alveolar gap, and the caudal septal and columellar deformity is quite pronounced. (b) Expected underlying cartilaginous deformity on the cleft side and significant asymmetry due to the “tilted tripod” theory. UCLND, unilateral cleft lip nasal deformity.

**Figure 10:**
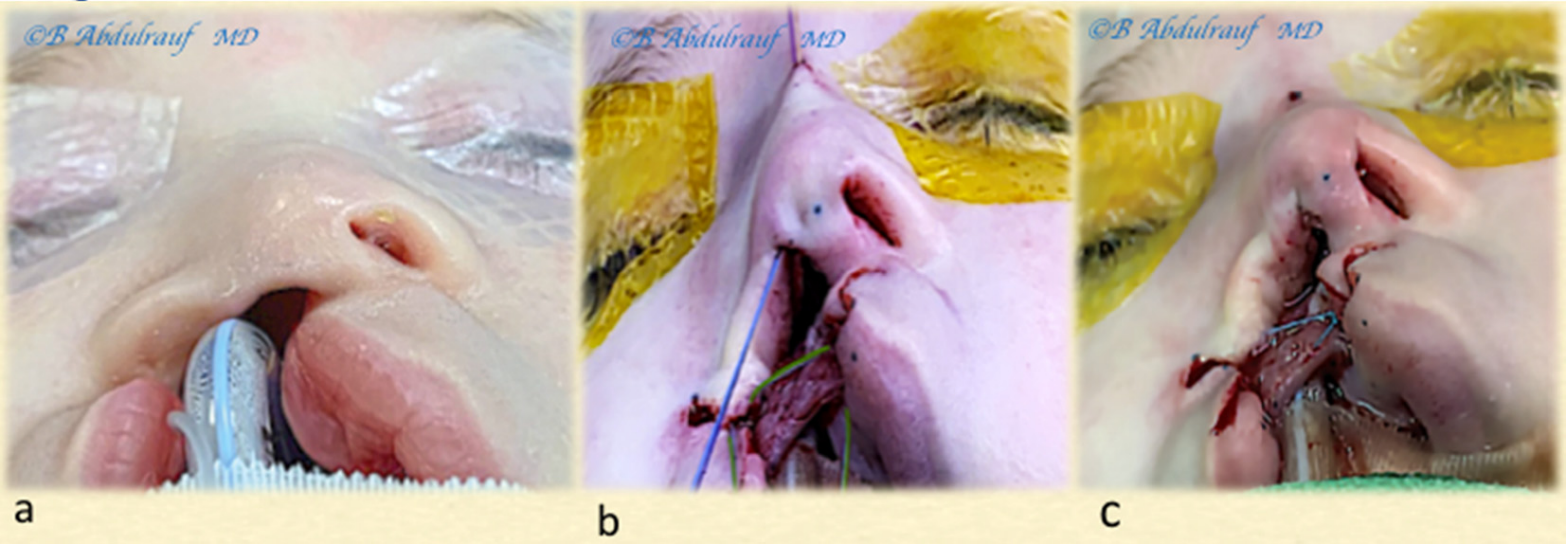
Case 2: (a) Intraoperative basal view. (b) The three key sutures: cinch Ethibond suture holding alar fibrofatty tissue to the premaxillary periosteum, transdomal PDS suture, and cantilever-lifting Vicryl thread seen coming out at the radix. All three sutures have been tied beginning with the cinch, followed by the transdomal, and finally the cantilever thread. (c) The nose has been fully reconstructed prior to and independent of the lip repair.

**Figure 11:**
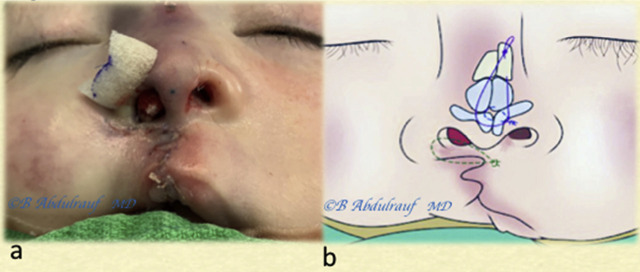
Case 2: A single bolster suture used at the alar rim (usually removed in 48 h). (a) The lip has been repaired; the fact it was mildly deficient in height was recognized. (b) Author’s simulation of nasal reconstruction and the three key sutures.

We often place bolster suture(s) as support for the dead space, usually to the alae. This reduces any minor hematomas and hence helps in reducing fibrosis. These are removed within 48 h (Figures 6, 11).

## Results

In total, nine patients were subjected to the nasal cantilever technique (NCT) as the only surgical intervention to the nose and lip up to their last follow-up. The first two cases were recent and were mostly performed as a demonstration with short follow-up. Age at the time of surgery ranged from 4 months to two years. Photographs of the results at follow-up were taken anywhere between 3 months and 10 years postoperatively (Table 1).

**Table 1:** Summary of the nine cases who were subjected to the NCT.

Case	Type of cleft	Age at surgery	Postop. follow-up period	Figure #
1.	BCLND *	14 M.	6 M.	2–8
2.	UCLND *	4 M.	4 M.	9–12
3.	BCLND	6 M.	1 Y.	13
4.	BCLND	8 M.	2 Y.	14
5.	UCLND **	18 M.	6 Y.	15
6.	UCLND	11 M.	6 Y.	16
7.	UCLND	5 M.	10 Y.	17
8.	UCLND	2 Y.	10 Y.	18
9.	UCLND	8 M.	10 Y.	19

**Single stage, cleft lip, and palate surgery

*Technique demonstration case

M = months; Y = years; UCLND, unilateral cleft lip nasal deformity; BCLND, bilateral cleft lip nasal deformity.

In terms of complications, in case 3, we encountered an issue with the alar cinch suture, a stitch granuloma. It was pulled out under conscious sedation.

## Discussion

### Approach to the lip

It was interesting to learn from a mentor like Dr. Hugh Thompson. This seems to be the norm; there is nothing wrong in doing a hybrid operation for the cleft lip, and nobody would object to an expert of being innovative [6].

Our first mentor, Dr. Miroslaw Stranc, who has extensively worked on lip function studies [7], is a strong believer in the rotation advancement technique and has often expressed, *“If you end up by doing clefts, your mistakes are going to grow with you!”*

Later on, as the first fellow with Dr. D. Fisher, I was influenced by his concept of the anatomic subunit principle, which was undergoing a prospective study at that time [8]. Later, with close to 20 years of performing cleft repairs, we found ourselves having adopted a few modifications from here and there as well. When we decide to perform rotation advancement, we usually use Noordhoff’s modification [9], [10].

Regarding a bilateral cleft, we tend to use a few concepts from both, the Millard and Mulliken techniques, but without narrowing the philtrum to near normal dimensions at the primary surgery. We disagree on discarding skin in infancy.

### Historic review of approaches to the nose

It is interesting that not too long ago surgeons began giving more serious consideration to the early cleft nose repair approach [11].

Historically, different suturing techniques have been suggested and described to secure the cartilages surgically dissected and freed at the dome area as well as cephalically [12].

Mattress sutures were used by Tajima and Masaru [13] in 1977 to secure the repositioned lower lateral cartilages, holding the lower lateral cartilages to the triangular cartilages as part of their described approach to secondary correction of the cleft nose. Kernahan et. al. [3] presented their results using the same technique as Tajima, who then presented the long-term results of the original approach with some additions [14].

After undermining the nasal skin, McComb [15] used mattress sutures to reposition the nasal cartilages, securing them externally with bolster sutures. The mattress sutures depend on dermal resistance to maintain their traction and need to be removed in approximately 5 days. They demonstrated the technique initially in UCLND [15] and later presented their long-term follow-ups in both unilateral and bilateral clefts [16], [17].

Besides the rim incisions, Stenstrom [18] added a small external incision on the dorsum to lift the affected alar cartilages and to secure them to the septal cartilage with nonabsorbable sutures.

### Current literature on cleft nasal deformity

Major authorities on cleft care and craft in the current era indicate that nasal deformity is the stigma that most likely remains clearly visible despite vigorous and repeated attempts at correction [4]. Due to several factors involved in the nasal cleft pathoanatomy, the deformity is not amenable to correction at the index operation [2]. It is easier to obtain results in the symmetric bilateral cleft lip compared to unilateral, and the latter requires more revisions [4]. Cleft lip repair is primarily a nasal surgery [1].

With all due respect, the old message or classic teaching that successful treatment of the bilateral complete cleft and palate can be the most difficult task [19], [20] is a misconception; reconstructing a double defect is not challenging. The tilted tripod theory applicable specifically to unilateral clefts makes them far more challenging from the nasal point of view and over the long term [5].

### Our technique, the NCT

The Nasal Cantilever (or Nasal lift technique) described here repositions the cartilages and soft tissues in a desired and overcorrected position after they have been completely freed, and holds that position internally to a fixed base, the periosteum (Figures 4c, 6b, 5b, 5d, 11b). The choice of the loop suture material was based on it being somewhat elastic and braided with good knot quality and absorbable within a reasonable time. We did not want to use a thread that would tear the tissues and defeat the purpose. Most of the suspension techniques or cable sutures anywhere depend on the creation of fibrous bands that would eventually replace the suture, similar to a scaffold.

Early in our practice, we were exclusively trained in open tip rhinoplasty. However, when dealing with infants, preschool children, and teenagers, one should logically aim for interceptive procedures.

Almost all of these children will someday consider definitive rhinoplasty with a proper open technique. The presence of columellar scars and fibrosis from early life would hinder definitive long-term rhinoplasty. Therefore, we had to acquire technical skills in the closed technique. With a learning curve, it is possible to reshape the nose with entirely closed techniques [4], [22]. The procedure presented here barely utilizes the infracartilaginous incisions and two tiny needle-induced openings in the skin.

### The lip and nose “tug of war phenomenon”

The fact that primary correction requires simultaneous repair of the lip and nose raises a question: will one of them need to be compromised?

Many surgeons would have good lip results but less than average nose results and vice versa. This is also why secondary “cleft rhinoplasty” in adults is not combined with lip revision. The nasal and lip units share borders and when there is clefting, it acts very much like a malformation*.*

Parameters such as presurgical orthopedic manipulation, strict collaborative programs, and compliance play a major role in the outcomes of cleft repairs. [2], [4], [21]. Cleft nose deformity correction, whether primary or secondary, has been a daunting task for many cleft surgeons to the extent that one author very humbly admitted that it has been impossible in their hands to correct this deformity [23].

In fact, very few “esthetic rhinoplasty” surgeons would like to deal with cleft noses. When an adult patient with this congenital anomaly consults a rhinoplasty surgeon, they would have very high expectations, simply because they consulted a cosmetic surgeon. The surgeon in turn knows that they would be unable to reach a result anywhere close to their average cosmetic rhinoplasties [2], [25].

When one is working with opposite vectors, it is difficult to reach optimum harmony, and a compromise on either side is expected*.* Tissues do their best to return their original state while combating the fibrosis created by the surgical intervention; hence, our analogy, “tug of war”, best explains the situation with primary or secondary corrections to the nose or lip (Figure 20).

**Figure 12:**
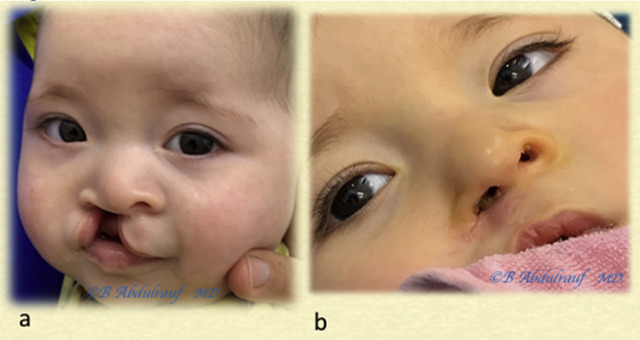
Case 2: (a) Used as an example for technique demonstration in UCLND at 4 months. (b) Five-month postoperative follow-up picture; the nose is maintaining its reconstructed shape. Lip outcome was somewhat expected. Some scar hypertrophy is noted at the nasal sill. UCLND, unilateral cleft lip nasal deformity.

**Figure 13:**
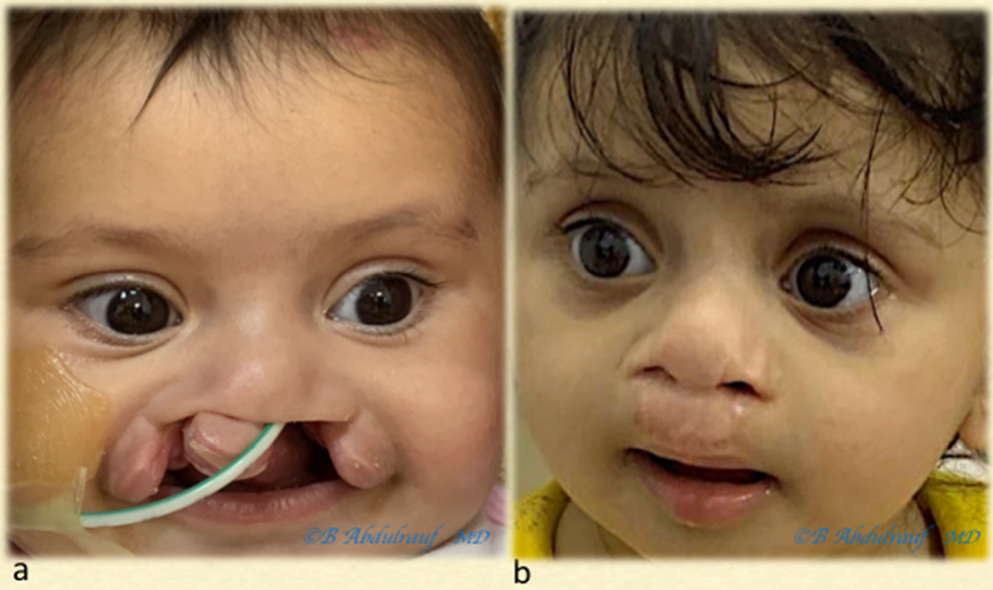
Case 3: (a) BCLND, severe and significant asymmetry, operated at 6 months. (b) One-year postoperative follow-up is shown. Although we use some maneuvers of Mulliken’s methods, we save most of the philtral skin at this age. BCLND, bilateral cleft lip nasal deformity.

**Figure 14:**
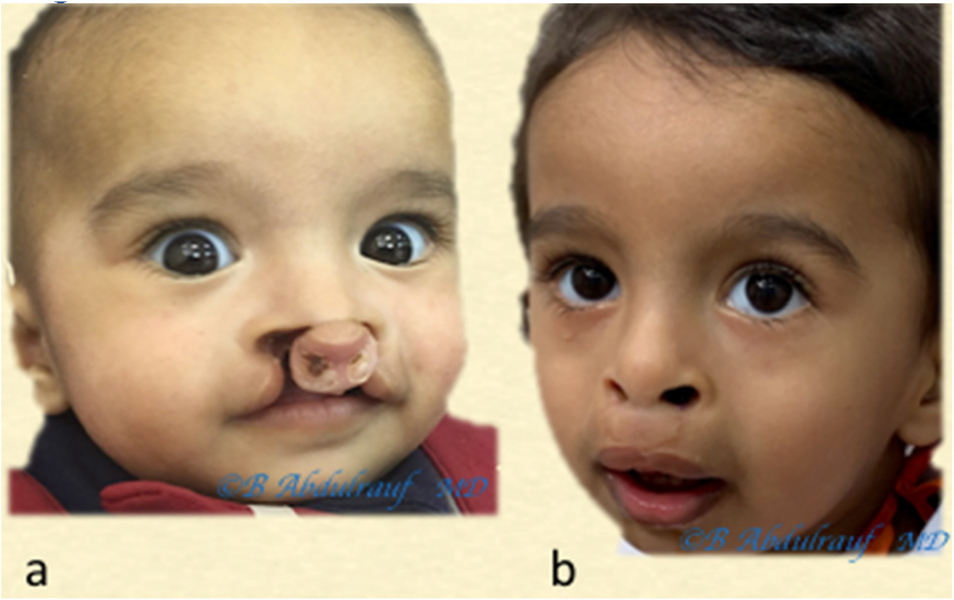
Case 4: (a) BCLND, like many other cases, the columella here has not formed; the was patient operated at the age of 8 months. (b) Follow-up picture at 2 years postoperatively. As usual, we do not discard any philtral skin at this age. Although the original anomaly is severe but also quite symmetric, the long-term esthetic outcome in this case can be predicted to be very good. This is contrary to older beliefs on BCLND. BCLND, bilateral cleft lip nasal deformity.

**Figure 15:**
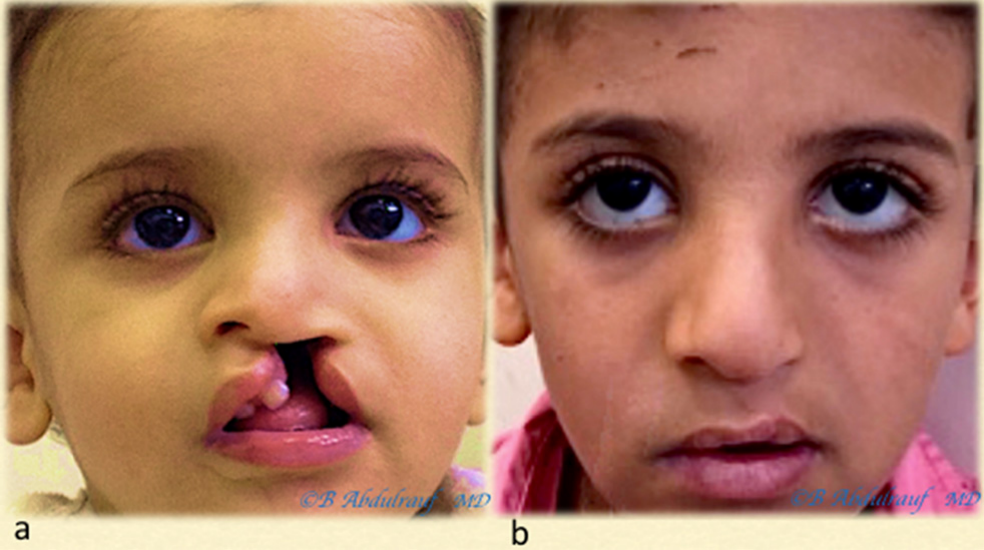
Case 5: (a) UCLND, this child was operated at 18 months as a single stage for cleft palate and UCLND. (b) Follow-up picture at 6 years postoperatively. UCLND, unilateral cleft lip nasal deformity.

**Figure 16:**
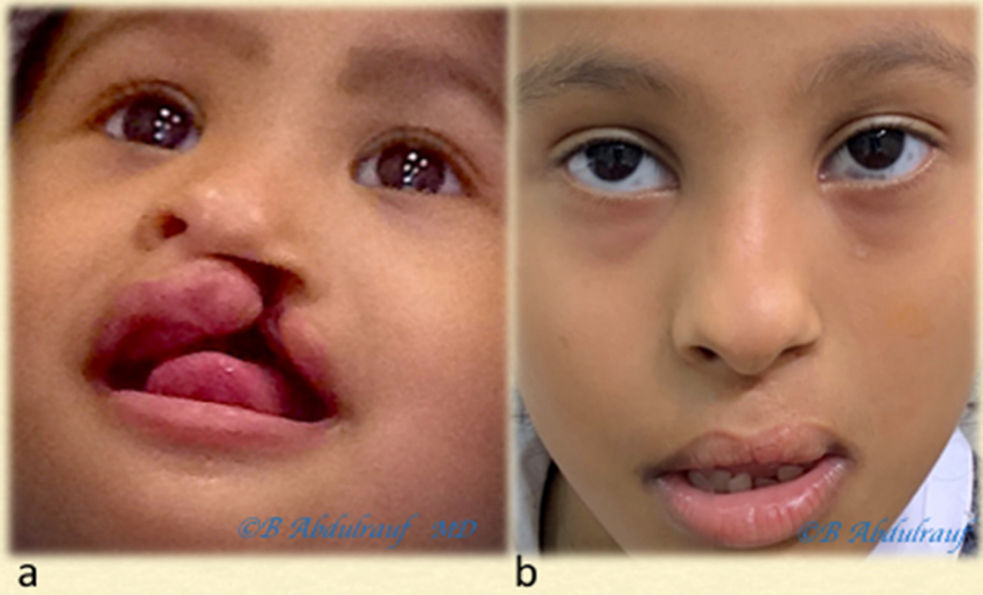
Case 6: (a) UCLND, operated at 11 months. (b) Follow-up picture at six years postsurgery. UCLND, unilateral cleft lip nasal deformity.

**Figure 17:**
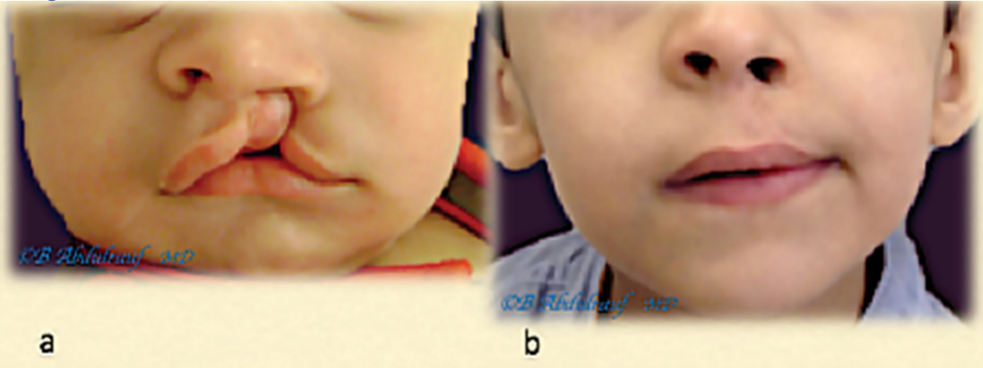
Case 7: (a) UCLND, operated at 5 months. (b) Follow-up picture at 10 years postsurgery. UCLND, unilateral cleft lip nasal deformity.

**Figure 18:**
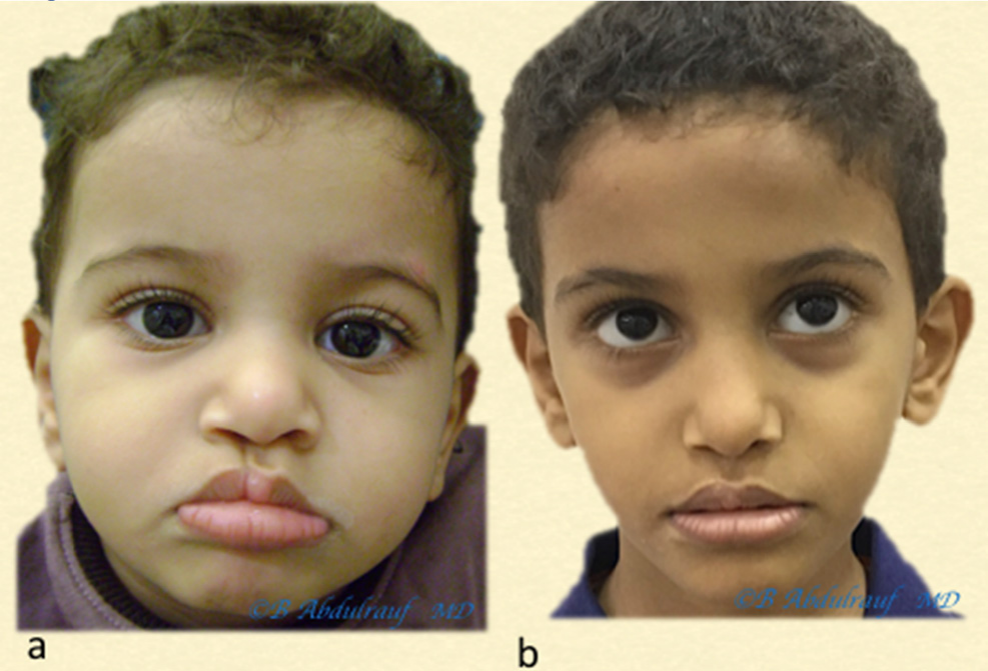
Case 8: (a) UCLND, “form fruste” type, was operated at two years. (b) Follow-up picture at 10 years postsurgery. UCLND, unilateral cleft lip nasal deformity.

**Figure 19:**
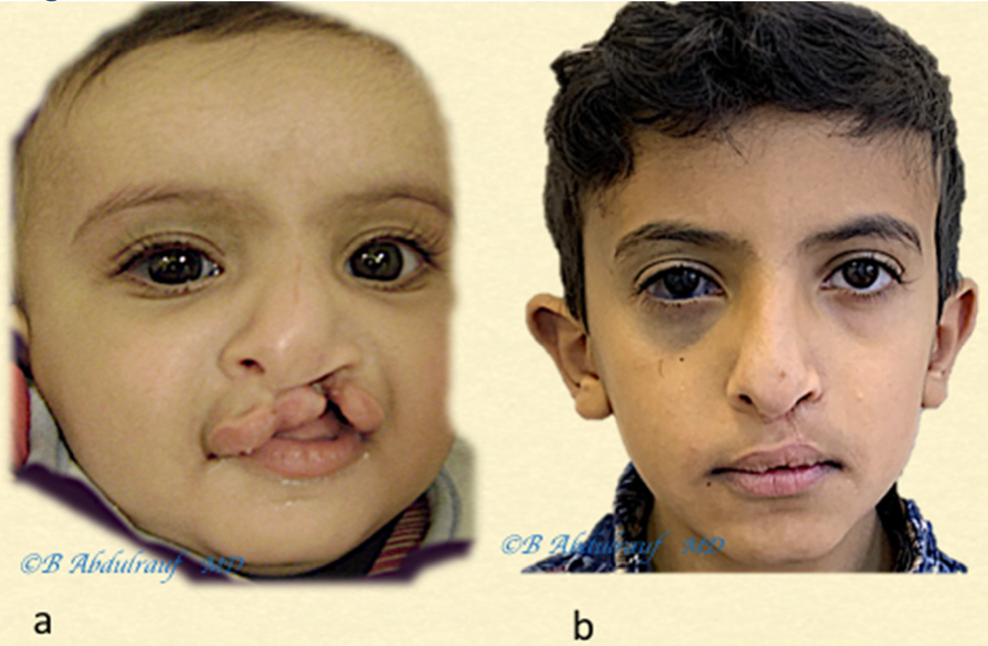
Case 9: (a) UCLND, operated at 8 months. (b) Follow-up picture at 10 years postsurgery; the lip has mild shortening, which might require revision. The Patient also has nevus of Ota, right orbit. UCLND, unilateral cleft lip nasal deformity.

**Figure 20:**
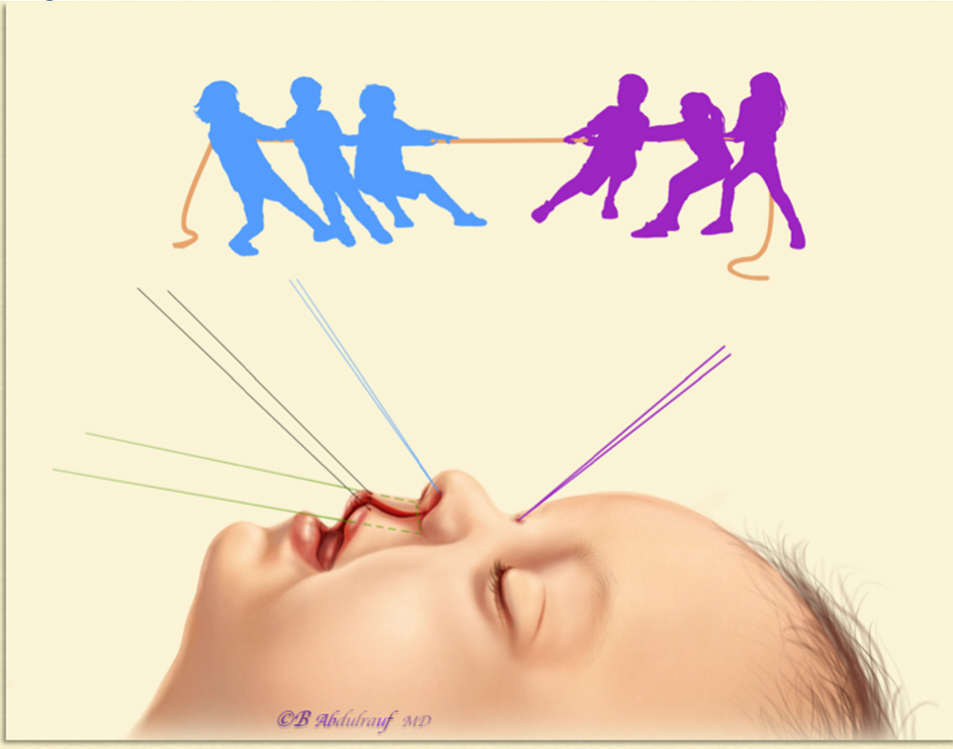
The nasal and lip units share borders; when there is a cleft, it tends to act as a malformation. Simultaneous repair of both, whether in childhood or secondarily, means attempting to recruit tissues with opposite vectors. Tissues will resist due to their inherent memory, and compromise of results on either side is probable. Hence, the analogy “*Tug of War”* phenomenon.

The cantilever concept in the nose is well known, originally of Converse and Millard, where they described a cantilever bone graft secured to the radix in reconstructing the dorsum [24].

Cleft lip nasal deformity has unique pathology [2]. The cartilages and skin tend to maintain their memory, and it falls mostly under the domain of reconstructive surgeons who are used to facing challenges and are expected to be creative [25], [26].

We believe the NCT method that lifts the whole nasal collapsing “tent” and holds it onto a fixed base (the nasion) is a new concept and promises to be the ultimate corrective approach to the nasal “pathoanatomy” in children with cleft nose and lip deformity.

## References

[1] Mulliken JB . Primary repair of bilateral complete cleft lip and nasal deformity. Plast Reconstr Surg 2001;108:181–94. 10.1097/00006534-200107000-00028. 11420522

[2] Fisher MD , Fisher DM , Marcus JR . Correction of the cleft nasal deformity: from infancy to maturity. Clin Plast Surg 2014 Apr;41:283–99. 10.1016/j.cps.2014.01.002. 24607195

[3] Kernahan DA , Bauer BS , Harris GD . Experience with the Tajima procedure in primary and secondary repair in unilateral cleft-lip nasal deformity. Plast Reconstr Surg 1980;66:46–53. 10.1097/00006534-198007000-00009. 7394046

[4] Mulliken J . Repair of bilateral complete cleft lip and nasal deformity-state of the art. Cleft Palate Craniofacial J 2000;37:342–7. 10.1597/1545-1569_2000_037_0342_robccl_2.3.co_2. 10912711

[5] Hogan VM , Converse JM . Secondary deformities of unilateral cleft lip and nose. In: Grabb WC , Rosentein SE , Bzoch KR , editors. Cleft lip and palate. Boston: Little Brown and co; 1971:245.

[6] Thomson H.G . Unilateral cleft lip repair. Operat Tech Plast Reconstr Surg Aug. 1995;2:175–81.

[7] Fogel M.L. , Stranc M.F . Lip function: a study of normal lip parameters. BJPS 1984;37:542–9. 10.1016/0007-1226(84)90147-4. 6498395

[8] Fisher D . Unilateral cleft lip repair: an anatomic subunit sub unit approximation technique. Plast Reconstr Surg 2005;116:61–71. 10.1097/01.prs.0000169693.87591.9b. 15988248

[9] Millard D.R . Refinements in rotation advancement cleft lip technique. Plast Reconstr Surg 1964;33:26–3. 10.1097/00006534-196401000-00003. 14104544

[10] Noordhoff MS . Reconstruction of vermilion in unilateral and bilateral cleft lips 1984:52–60. 10.1097/00006534-198401000-00011. 6691075

[11] Millard DR . Earlier correction of the unilateral cleft lip nose. Plast Reconstr Surg 1982;70:64–73. 10.1097/00006534-198207000-00014. 7089109

[12] Converse JM , . Secondary deformities of the cleft lip, cleft lip and nose, and cleft palate. In: Converse JM , McCarthy JG , editors. Reconstructive plastic surgery 4 Philadelphia: WB Saunders; 1977:2165.

[13] Tajima S , Masaru M . Reverse-U incision for secondary repair of cleft lip nose. Plast Reconstr Surg 1977 Aug;60:256–61. 10.1097/00006534-197708000-00013. 887664

[14] Tajima S . Follow up results of the unilateral primary cleft lip operation with special reference to primary nasal correction by the author’s method. Facial Plast Surg 1990:97–104. 10.1055/s-2008-1064669. 2132238

[15] McComb H . Treatment of the unilateral cleft lip nose. Plast Reconstr Surg May 1975;55:596–601. 10.1097/00006534-197505000-00010. 1144536

[16] McComb HK , Coghlan BA . Primary repair of the unilateral cleft lip nose: completion of a longitudinal study. Cleft Palate-Craniofacial J 1996 Jan;33:23–30. 10.1597/1545-1569(1996)033<0023:protuc>2.3.co;2 discussion 30-1. 8849855

[17] McComb H . Primary repair of the bilateral cleft nose: 10-year review. Plast Reconstr Surg May 1986;77:709–13. 10.1097/00006534-198605000-00001 701-706. 10.1097/00006534-198605000-00001 3517905

[18] Stenstrom SJ . Correction of cleft lip nasal deformity; a refinement of an older method. Plast Reconstr Surg 1977;59:675. 10.1097/00006534-197705000-00009. 850704

[19] Manchester W.M . The repair of bilateral cleft lip and palate. BJPS 1965;52:878–82. 10.1002/bjs.1800521111. 5842977

[20] Brown JB , McDowell F , Byars LT . Double clefts of the lip. Surg. Gynaecolog. Obstet. 1974;85:20. 20249242

[21] Grayson BH , Cutting CB . Presurgical naso- alveolar orthopedic molding in primary correction of the nose. Lip and alveolus of infants born with unilateral and bilateral clefts. Cleft Palate Craniofacial. J 2001:193–8. 10.1597/1545-1569(2001)038<0193:pnomip>2.0.co;2. 11386426

[22] Tibbets J . Shaping and positioning the nasal tip without structural disruption: a new systematic approach. Plast Reconstr Surg: July 1994;94:61–77. 10.1097/00006534-199407000-00006. 8016254

[23] Randall P . History of cleft lip nasal repair. Cleft Palate Craniofacial. J. 1992;29:527–30. 10.1597/1545-1569(1992)029<0527:hoclnr>2.3.co;2. 1450193

[24] Converse JM , editor. Reconstructive plastic surgery, 2nd ed. Philadelphia: Saunders; 1977.

[25] Abdulrauf B . The Reconstructive plastic surgeon vs the aesthetic plastic surgeon: perspective. Int. J. of Surgery and Clinical practice 2 Pubtexto publishers open- access; 2020:1–2.

[26] Rohrich RJ , Sullivan D . So do you want to be like Leonardo da Vinci or Michaelangelo?. Which one are you?. Plast Reconstr Surg 2011 Dec;128:1309–11. 10.1097/prs.0b013e31820edbf6. 22094748

