# Supplementary material for: The nasal cantilever technique in children undergoing primary cleft lip surgery: novel concepts and review
Source: Innov Surg Sci. 2020 Sep 7;5(1-2):53–62. doi: 10.1515/iss-2020-0016 (PMC7798302; doi:10.1515/iss-2020-0016)
Supplement: Supplementary file 1 [file iss-5-20200016-s001.pdf]

Reviewer Assessment

Badr Abdulrauf: The Nasal Cantilever technique in Children undergoing Primary Cleft Lip surgery: Novel Concepts and Review.

Reviewers' Comments to Original Submission

Reviewer 1: anonymous

Date received: 28-Jun-2020
Reviewer recommendation: **Return to author for major modifications**
Reviewer overall scoring: **Medium**

Assessment Form scores: 5 = High/Yes; 3 = Medium/Adequate; 1 = Low

Is the subject area appropriate for the journal	5				
Does the title clearly reflect the paper's content?	5				
Does the abstract clearly reflect the paper's content	5				
Do the keywords clearly reflect the paper's content?	5				
Does the introduction present the problem clearly?			3		
Are the results/ conclusions justified?			3		
How comprehensive and up-to-date is the subject matter presented?			3		
How adequate is the data presentation?			3		
Are units and terminology used correctly?	5				
Is the number of cases adequate?				2	
Are the experimental methods/ clinical studies adequate?		4			
Is the length appropriate in relation to the content?					1
Does the reader get new insights from the article?		4			
Please rate the practical significance.		4			
Please rate the accuracy of methods.		4			
Please rate the statistical evaluation and quality control.				2	
Please rate the appropriateness of the figures and tables.			3		
Please rate the appropriateness of the references.	5				
Please evaluate the writing style and use of language.		4			
Please judge the overall scientific quality of the manuscript.	5				
Are the methods used worthy of reproduction in greater deal?	yes				

Comments to author: An excellent concept and magnificent drawings. Regrettably, clinical photos are not of the same standard. I think that presentation of images, four in line is distracting. A combination of a clinical photo and an explanatory

drawing would serve better. Of course more cases would help, but this is a presentation which introduces a new approach to a difficult problem and is worth presenting.

Reviewer 2: anonymous

Date received: 16-Jul-2020
Reviewer recommendation: **Return to author for minor modifications**
Reviewer overall scoring: **High**

Assessment Form scores: 5 = High/Yes; 3 = Medium/Adequate; 1 = Low

Is the subject area appropriate for the journal		4			
Does the title clearly reflect the paper's content?		4			
Does the abstract clearly reflect the paper's content	5				
Do the keywords clearly reflect the paper's content?	5				
Does the introduction present the problem clearly?		4			
Are the results/ conclusions justified?			3		
How comprehensive and up-to-date is the subject matter presented?	5				
How adequate is the data presentation?		4			
Are units and terminology used correctly?		4			
Is the number of cases adequate?			3		
Are the experimental methods/ clinical studies adequate?			3		
Is the length appropriate in relation to the content?		4			
Does the reader get new insights from the article?		4			
Please rate the practical significance.		4			
Please rate the accuracy of methods.			3		
Please rate the statistical evaluation and quality control.			3		
Please rate the appropriateness of the figures and tables.		4			
Please rate the appropriateness of the references.	5				
Please evaluate the writing style and use of language.		4			
Please judge the overall scientific quality of the manuscript.		4			
Are the methods used worthy of reproduction in greater deal?	yes				
Would you be willing to review a revision of this manuscript?	no				

Comments to author: The authors present a sound study about a novel suspension technique by Lifting the entire cartilaginous structure of the nose from infra tip region, with a loop suture and being secured onto the periosteum in cleft patients. They present 9 cases with a follow up time of "months to years" and concluded a new method that lifts the whole nasal collapsing “Tent” and holds it into a fixed base (the nasion) in children with cleft Nasal-Lip deformity. Despite the small sampe sice and no objective Facts this article might be an interesting additive for surgeons Performing these operations. However, I have one Annotation. a few spelling disorders and mistakes are to be corrected.

Authors' Response to Reviewer Comments

Date received: 20-Jul-2020

Response to reviewer 1

Many thanks. We have revised the figures. Majority of the figures now have 2 segments only. The figure with 4 in a row was changed to avoid distraction. The only figure which we had to keep same is figure 5 which shows before after clinical results on operating table and with artist's illustration underneath. A number of clinical pictures were also replaced with better resolution ones. I hope they are much more satisfactory now. Best Regards.

Response to reviewer 2

Many Thanks. We submitted the manuscript to "Wolters Kluwer" and had it fully edited for grammar and language issues. A certificate has been attached as well. I believe this step was worth it considering the efforts already taken for this work. Best Regards.

Reviewers' Comments to Revised Submission

Reviewer 1: anonymous

Date received: DD-MM-2020
Reviewer recommendation: **Return to author for minor modifications**
Reviewer overall scoring: **High**

Assessment Form scores: 5 = High/Yes; 3 = Medium/Adequate; 1 = Low

Is the subject area appropriate for the journal	5				
Does the title clearly reflect the paper's content?	5				
Does the abstract clearly reflect the paper's content	5				
Do the keywords clearly reflect the paper's content?	5				
Does the introduction present the problem clearly?	5				
Are the results/ conclusions justified?			3		
How comprehensive and up-to-date is the subject matter presented?			3		
How adequate is the data presentation?	5				
Are units and terminology used correctly?	5				
Is the number of cases adequate?				2	
Are the experimental methods/ clinical studies adequate?		4			
Is the length appropriate in relation to the content?		4			
Does the reader get new insights from the article?	5				
Please rate the practical significance.		4			
Please rate the accuracy of methods.	5				
Please rate the statistical evaluation and quality control.				2	
Please rate the appropriateness of the figures and tables.	5				

Please rate the appropriateness of the references.		4			
Please evaluate the writing style and use of language.					
Please judge the overall scientific quality of the manuscript.	5				
Are the methods used worthy of reproduction in greater deal?	yes				

Comments to author: A very good idea on how to tackle nasal deformity. Presentation excellent, especially the drawings. You need more cases and a long term, at least two years past puberty, follow up. If you can reduce number of illustrations.

Comments by the Editor-in-Chief to Revised Submission

All reviewer concerns raised were addressed satisfactorily. The manuscript may be published in the present stage.
